# Extracellular vesicles and viruses share a nuclear entry pathway in cancer and infection

**DOI:** 10.3389/fcell.2026.1796338

**Published:** 2026-03-30

**Authors:** Aurelio Lorico, Denis Corbeil

**Affiliations:** 1 College of Medicine, Touro University Nevada, Henderson, NV, United States; 2 Tissue Engineering Laboratories, Biotechnology Center (BIOTEC) and Center for Molecular and Cellular Bioengineering, Technische Universität Dresden, Dresden, Germany; 3 Tissue Engineering Laboratories, Medizinische Fakultät der Technischen Universität Dresden, Dresden, Germany

**Keywords:** endosome, exosome, HIV-1, intercellular communication, intracellular trafficking, microvesicle, nucleus, viral infection

## Introduction

The concept that small extracellular vesicles (sEVs; 30–200 nm in diameter) can deliver cargo to the nucleus has gained increasing attention in recent years. Several features of this process echo aspects of retroviral infection, even though EVs lack replicative capacity. These parallels have revived interest in the “Trojan exosome” hypothesis (Gould et al., 2003), which posits that sEVs and retroviruses exploit overlapping biogenesis routes and share key functional traits. However, the mechanism governing nuclear entry of extracellular particles is far from settled. Recent evidence points to coordinated interactions between endosomes and the nuclear envelope in both events, raising the possibility that a common nuclear gateway may facilitate both intercellular communication and pathogenic entry.

### sEVs as mediators of cellular communication

sEVs are widely distributed and have been identified in numerous biological fluids. They are secreted as exosomes, originating from the endocytic pathway, or microvesicles (also termed ectosomes), budding directly from the plasma membrane (van Niel et al., 2018; Welsh et al., 2024). sEVs mediate both local and systemic intercellular communication through paracrine signaling, playing fundamental roles in cell development and homeostasis.

The sEV membrane enables interactions with cell-surface receptors, whereas the vesicular lumen contains source cell-specific molecules such as RNAs, proteins and other intercellular messengers. In disease, sEVs can disseminate pathogenic cargoes. For example, in cancer, tumor cell-derived sEVs convey oncogenic and immunomodulatory factors to stromal and immune cells, thereby reconfiguring the tumor microenvironment, promoting metastasis, and suppressing anti-tumor immunity (Kalluri and McAndrews, 2023).

### Organelle cross-talk in EV trafficking

How sEVs deliver consequential signals within recipient cells remains an area of active investigation. In the acidic tumor microenvironment, sEVs may fuse with the plasma membrane, releasing their contents directly into the pericellular cytoplasm. More commonly, however, they are internalized through diverse endocytic pathways, after which cargo can access deeper cytoplasmic regions following endosomal escape (van Niel et al., 2018; Ribovski et al., 2023). A key question is how sEV-derived molecules achieve concentrations sufficient to exert biological activity within the large cytoplasmic volume of recipient cells. Growing evidence suggests that this challenge may be addressed through coordinated organelle cross-talk. Interactions between sEV-containing endosomes and intracellular organelles may position EV cargoes near subcellular sites where they can exert maximal functional impact.

For instance, interactions between sEV-containing endosomes and the endoplasmic reticulum (ER) have been shown to position sEV-derived mRNAs (or miRNAs) near active translation sites, thereby enhancing their functional impact on protein synthesis (Heusermann et al., 2016). Such spatial organization may amplify weak signals and restrict responses to specific subcellular domains. From this perspective, intracellular routing of EV cargo is unlikely to rely solely on passive diffusion but instead appears to involve a spatially coordinated trafficking network that shapes the specificity and timing of EV-mediated signaling.

### Nucleus as a potential hub of EV intercellular signaling

Recent observations further extend this spatiotemporal concept, suggesting that a fraction of molecules (proteins and nucleic acids) derived from endocytosed sEVs can reach the nuclear compartment in both healthy and cancer cells (Cai et al., 2013; Read et al., 2017; Rappa et al., 2017; Seltmann et al., 2024). These findings raise the intriguing possibility that nuclear trafficking may represent an additional regulatory layer linking sEV-mediated communication directly to gene expression.

For example, Seltmann and colleagues demonstrated that sEV-associated chloride channel accessory 2, upon internalization and nuclear translocation in recipient cells, interacts with β-catenin and promotes Wnt target gene expression (Seltmann et al., 2024). Similarly, our previous work showed that cancer-derived sEVs modulate the transcriptional activity of mesenchymal stromal cells, notably downregulating genes involved in inflammatory responses (Rappa et al., 2017). Despite these observations, the minimum cargo concentration required to elicit functional transcriptional changes remains unclear. The nuclear fate of EV cargo therefore represents an emerging frontier in EV biology. Elucidating these pathways could reveal previously unrecognized regulatory mechanisms governing tissue homeostasis, development, and disease progression.

How endosomal pathways intersect with nuclear entry mechanisms remains largely unexplored (Chaumet et al., 2015; Rappa et al., 2017), yet this interface likely represents a decisive junction between extracellular cues and transcriptional responses. One proposed mechanism involves direct interactions between early endosomes and the nuclear envelope, a phenomenon previously described for the nuclear delivery of several receptors and extracellular ligands (Chaumet et al., 2015). This pathway entails the transport, docking, and fusion of endosomes with the outer nuclear membrane and requires nuclear pores, SUN1/2 proteins, and the Sec61 translocon complex, which facilitates protein translocation from the inner nuclear membrane into the nucleoplasm (Chaumet et al., 2015). Although it remains unclear whether sEVs utilize this machinery, these mechanistic parallels highlight the potential flexibility of nuclear import pathways in responding to extracellular signals.

A related intracellular transport mechanism involving late endosomes has been proposed to explain the nuclear delivery of sEV cargo. In this model, endosomes carrying sEVs accumulate in the perinuclear region at the microtubule-organizing center (Rappa et al., 2017). A subset of these late endosomes subsequently translocate into type-II nuclear envelope invaginations (NEIs) (Malhas et al., 2011) in a microtubule-dependent manner (Santos et al., 2018). This process is supported by the presence of tubulin within NEIs and by experiments showing that treatment with nocodazole, a microtubule-inhibiting agent, impairs the ingress of Rab7^+^ late endosomes into NEIs (Santos et al., 2018). This pathway was termed “*Spathasome”*, reflecting the resemblance of accumulated late endosomes within narrow NEIs to a sword (*spatha*) sheathed in its scabbard (Rappa et al., 2017). NEIs may provide a protected environment that shields sEV-containing endosomes from lysosomal degradation while granting access to deeper nuclear-associated regions, including the nucleolus, where the type-II NEIs frequently terminate (Zhuang et al., 2024). Whether the endosomes that enter NEIs do so through specific endocytic mechanisms remains to be determined.

Within these confined NEIs, sEV cargo may escape acidified endosomes through the back-fusion of internalized sEVs with the limiting endosomal membrane (Perrin et al., 2021).

Consistent with this possibility, sEV–endosome fusion events have been recently observed in the vicinity of the nuclear compartment (van den Ende et al., 2026). This proximity potentially grants sEV cargo access to cytoplasmic targets exported from the nucleus, or enables their entry into the nucleus through nuclear pores. Pharmacological inhibition of importin-β1 using importazole impairs nuclear accumulation of EV-derived cargo, suggesting that active nuclear import mechanisms are involved (Rappa et al., 2017; Santos et al., 2018). The association of importin-β1 with sEVs may therefore have functional significance (Rappa et al., 2017), an issue that warrants further investigation.

The possibility that intact, very small EVs (<40 nm) could penetrate the nucleus remains unresolved (Seltmann et al., 2024). If confirmed, such a pathway would likely require rupture of the endosomal limiting membrane rather than fusion (reviewed in Ref. (Varkouhi et al., 2011)), as such disruption would be necessary for intact vesicles to access the nuclear compartment.

### The VOR complex as a molecular scaffold

A key molecular scaffold appears to coordinate this NEI-based trafficking route. A tripartite complex, termed “VOR” complex, composed of VAP-A at the outer nuclear membrane, the cytoplasmic oxysterol-binding protein ORP3, and the small GTPase Rab7 on late endosomes, creates a physical bridge that enables docking of late endosome at the outer nuclear envelope and facilitates their entry into NEIs (Santos et al., 2018; Santos et al., 2021). The proximity of these three components within the NEIs was demonstrated using a fluorescence resonance energy transfer–acceptor photobleaching approach (Santos et al., 2018; Santos et al., 2021).

Silencing VAP-A or ORP3 prevents Rab7^+^ late endosomes from entering NEIs and markedly reduces nuclear EV-derived cargo (Santos et al., 2018; Santos et al., 2021). These observations argue against the possibility that nuclear EV signals arise from imaging artifacts associated with confocal z-stack three-dimensional reconstruction and instead support the existence of an active trafficking pathway linking endosomes to the nuclear compartment, notably NEIs. They also indicate that nuclear delivery does not occur solely through classical mechanisms such as cytoplasmic diffusion followed by nuclear pore import or passive entry during nuclear envelope disassembly during mitosis.

The VOR complex appears distinct from previously described ER-associated VAP-A-dependent endosomal positioning mechanisms operating within the cytoplasmic compartment, which are regulated through cholesterol-dependent interactions with another oxysterol-binding protein, ORP1L (Rocha et al., 2009; van der Kant et al., 2013). The absence of ORP1L from NEIs suggests that different VAP-A–oxysterol-binding protein assemblies regulate distinct intracellular trafficking routes (Rappa et al., 2017; Santos et al., 2018). Notably, VAP-A–ORP3 interactions have also been implicated in the regulation of ER–plasma membrane contact sites together with the small GTPase R-Ras (Lehto et al., 2008).

Taken together, these findings suggest that nuclear-associated endosomal trafficking constitutes a regulated, spatially organized pathway that may influence EV-mediated intercellular communication and downstream cellular responses.

### Shared nuclear axis of sEVs and HIV-1

The reprogramming of transcriptional landscapes mediated by sEVs is reminiscent of strategies used by enveloped viruses that exploit host nuclear machinery to complete their life cycle. Early studies suggested that HIV-1 enters cells through direct fusion with the plasma membrane (Stein et al., 1987). However, subsequent work demonstrated that endocytosis can also serve as a relevant route (Miyauchi et al., 2009; Sharma et al., 2023). Like sEVs, retroviral particles are nanosized, incorporate host cell components, and rely on cellular machineries for their biogenesis, packaging, release, and uptake, as highlighted by the “Trojan exosome” hypothesis (Gould et al., 2003). This conceptual overlap raises the possibility that retroviruses hijack pre-existing intracellular trafficking routes used by sEVs to access the nuclear compartment.

Recent evidence supports this idea. HIV-1 can deliver viral components, such as integrase, to the nucleus of infected cells via late endosomes and NEIs, leading to productive infection (Santos et al., 2023). This mechanism was demonstrated using HIV-1 pseudotyped with vesicular stomatitis virus G or the native Env protein in HeLa cells and CD4^+^ T cells, and was supported by fluorescence microscopy and biochemical analyses. Notably, this process also depends on VAP-A and ORP3, suggesting that HIV-1 and sEVs may exploit a common nuclear entry pathway (Santos et al., 2023).

During CD4^+^ T-cell activation, protein kinase C-mediated phosphorylation of ORP3 promotes assembly of the VOR complex and the formation of NEIs, events that appear essential for nuclear delivery of viral components and productive infection (Santos et al., 2023). In quiescent CD4^+^ T cells, where ORP3 is not phosphorylated, the VOR complex does not form. Interestingly, activated CD4^+^ T cells, which normally lack NEIs, can generate these structures *de novo* during infection, suggesting a “push-in” mechanism in which late endosomes carrying viral particles promote their own entry into the nascent NEIs (Santos et al., 2023). Such a mechanism could also explain the sEV-dependent induction of NEIs (Rappa et al., 2017; Santos et al., 2021) and may contribute to the abundance of these structures in cancer cells, where circulating EV levels are elevated. Whether microtubule-dependent motor proteins participate in this process during infection remains unclear, although nocodazole treatment has been shown to impede VOR complex formation in infected, activated CD4^+^ T cells (Santos et al., 2023). The structural properties of NEI-associated nuclear pores remain largely unknown. Remodeling of the nuclear envelope within NEIs may increase the pore permeability (Rappa et al., 2017; Santos et al., 2021), allowing the passage of large macromolecules (>40 nm). Supporting this, recent studies show that the cone-shaped HIV-1 capsid (∼60 nm) can traverse nuclear pores by mechanically deforming them, suggesting NEI-associated pores may similarly accommodate large extracellular or viral particles (Zila et al., 2021; Kreysing et al., 2025). Finally, it is important to point out that this proposed new route of nuclear trafficking does not exclude the contribution of other pathways, notably direct fusion of viral particles with the plasma membrane and the action of HIV-1 accessory proteins, which may predispose the nuclear envelope to deformation.

Of note, because silencing VAP-A or ORP3 disrupts the nuclear communication axis exploited by both sEVs and HIV-1, the tripartite VOR complex represents a potential therapeutic target in pathologies where extracellular particles drive nuclear reprogramming of healthy cells in the tumor microenvironment or virally infected cells. Small-molecule inhibitors targeting the ORP3 sterol-binding pocket have been shown to block VOR complex assembly and impair both nuclear cargo transfer and NEI formation (Santos et al., 2021; Santos et al., 2023). However, targeting nuclear-endosomal trafficking could also affect normal cellular functions, and the translational implications of such interventions remain speculative (Carbone et al., 2024).

## Conclusion and future directions

The emerging view that sEVs and retroviruses may exploit a shared nuclear communication axis helps explain why viruses hijack the vesiculation machinery and incorporate sEV components into their infectious particles (Molnar et al., 2024). By co-opting sEV uptake and intracellular transport, retroviruses can traverse endosomal maturation steps that guide selected extracellular particles toward the nuclear envelope.

Despite these advances, this novel pathway requires further validation, and several fundamental questions remain unresolved. The molecular motors that direct late endosomes toward NEIs have yet to be identified, and the mechanisms governing NEI formation are still poorly understood. In addition, the structural and functional properties of nuclear pores within NEIs, and their potential remodeling during cargo transport, require further investigation.

From a translational perspective, the VOR complex represents a promising therapeutic target. Pharmacological disruption of ORP3-dependent interactions can inhibit assembly of the VOR complex, impair NEI formation, and limit nuclear cargo transfer. Targeting this pathway may therefore offer novel strategies for interfering with EV-driven tumor-stroma communication or preventing viral infection. Conversely, a deeper understanding of this trafficking route could inform the design of engineered vesicles capable of delivering therapeutic molecules directly to the nucleus.

## References

[B1] Cai J. Han Y. Ren H. Chen C. He D. Zhou L. (2013). Extracellular vesicle-mediated transfer of donor genomic DNA to recipient cells is a novel mechanism for genetic influence between cells. J. Mol. Cell. Biol. 5, 227–238. 10.1093/jmcb/mjt011 23580760 PMC3733418

[B2] Carbone D. Santos M. F. Corbeil D. Vistoli G. Parrino B. Karbanová J. (2024). Triazole derivatives inhibit the VOR complex-mediated nuclear transport of extracellular particles: potential application in cancer and HIV-1 infection. Bioorg. Chem. 150, 107589. 10.1016/j.bioorg.2024.107589 38941696

[B3] Chaumet A. Wright G. D. Seet S. H. Tham K. M. Gounko N. V. Bard F. (2015). Nuclear envelope-associated endosomes deliver surface proteins to the nucleus. Nat. Commun. 6, 8218. 10.1038/ncomms9218 26356418 PMC4579783

[B4] Gould S. J. Booth A. M. Hildreth J. E. (2003). The trojan exosome hypothesis. Proc. Natl. Acad. Sci. USA. 100, 10592–10597. 10.1073/pnas.1831413100 12947040 PMC196848

[B5] Heusermann W. Hean J. Trojer D. Steib E. von Bueren S. Graff-Meyer A. (2016). Exosomes surf on filopodia to enter cells at endocytic hot spots, traffic within endosomes, and are targeted to the ER. J. Cell Biol. 213, 173–184. 10.1083/jcb.201506084 27114500 PMC5084269

[B6] Kalluri R. McAndrews K. M. (2023). The role of extracellular vesicles in cancer. Cell 186, 1610–1626. 10.1016/j.cell.2023.03.010 37059067 PMC10484374

[B7] Kreysing J. P. Heidari M. Zila V. Cruz-Leon S. Obarska-Kosinska A. Laketa V. (2025). Passage of the HIV capsid cracks the nuclear pore. Cell 188, 930–943 e921. 10.1016/j.cell.2024.12.008 39826544

[B8] Lehto M. Mäyränpää M. I. Pellinen T. Ihalmo P. Lehtonen S. Kovanen P. T. (2008). The R-Ras interaction partner ORP3 regulates cell adhesion. J. Cell Sci. 121, 695–705. 10.1242/jcs.016964 18270267

[B9] Malhas A. Goulbourne C. Vaux D. J. (2011). The nucleoplasmic reticulum: form and function. Trends Cell Biol. 21, 362–373. 10.1016/j.tcb.2011.03.008 21514163

[B10] Miyauchi K. Kim Y. Latinovic O. Morozov V. Melikyan G. B. (2009). HIV enters cells *via* endocytosis and dynamin-dependent fusion with endosomes. Cell 137, 433–444. 10.1016/j.cell.2009.02.046 19410541 PMC2696170

[B11] Molnar S. M. Kim Y. Wieczorek L. Williams A. Patil K. A. Khatkar P. (2024). Extracellular vesicle isolation methods identify distinct HIV-1 particles released from chronically infected T-cells. J. Extracell. Vesicles 13, e12476. 10.1002/jev2.12476 38978287 PMC11231049

[B12] Perrin P. Janssen L. Janssen H. van den Broek B. Voortman L. M. van Elsland D. (2021). Retrofusion of intralumenal MVB membranes parallels viral infection and coexists with exosome release. Curr. Biol. 31, 3884–3893 e3884. 10.1016/j.cub.2021.06.022 34237268 PMC8445322

[B13] Rappa G. Santos M. F. Green T. M. Karbanová J. Hassler J. Bai Y. (2017). Nuclear transport of cancer extracellular vesicle-derived biomaterials through nuclear envelope invagination-associated late endosomes. Oncotarget 8, 14443–14461. 10.18632/oncotarget.14804 28129640 PMC5362417

[B14] Read J. Ingram A. Al Saleh H. A. Platko K. Gabriel K. Kapoor A. (2017). Nuclear transportation of exogenous epidermal growth factor receptor and androgen receptor *via* extracellular vesicles. Eur. J. Cancer. 70, 62–74. 10.1016/j.ejca.2016.10.017 27886573

[B15] Ribovski L. Joshi B. Gao J. Zuhorn I. (2023). Breaking free: endocytosis and endosomal escape of extracellular vesicles. Extracell. Vesicles Circ. Nucl. Acids. 4, 283–305. 10.20517/evcna.2023.26 39697985 PMC11648447

[B16] Rocha N. Kuijl C. van der Kant R. Janssen L. Houben D. Janssen H. (2009). Cholesterol sensor ORP1L contacts the ER protein VAP to control Rab7-RILP-p150 glued and late endosome positioning. J. Cell Biol. 185, 1209–1225. 10.1083/jcb.200811005 19564404 PMC2712958

[B17] Santos M. F. Rappa G. Karbanová J. Kurth T. Corbeil D. Lorico A. (2018). VAMP-Associated protein-A and oxysterol-binding protein-related protein 3 promote the entry of late endosomes into the nucleoplasmic reticulum. J. Biol. Chem. 293, 13834–13848. 10.1074/jbc.RA118.003725 30018135 PMC6130944

[B18] Santos M. F. Rappa G. Karbanová J. Fontana S. Bella M. A. D. Pope M. R. (2021). Itraconazole inhibits nuclear delivery of extracellular vesicle cargo by disrupting the entry of late endosomes into the nucleoplasmic reticulum. J. Extracell. Vesicles 10, e12132. 10.1002/jev2.12132 34429859 PMC8363911

[B19] Santos M. F. Rappa G. Karbanová J. Diana P. Cirrincione G. Carbone, D D. (2023). HIV-1-induced nuclear invaginations mediated by VAP-A, ORP3, and Rab7 complex explain infection of activated T cells. Nat. Commun. 14, 4588. 10.1038/s41467-023-40227-8 37563144 PMC10415338

[B20] Seltmann K. Hettich B. Abele S. Gurri S. Mantella V. Leroux J. C. (2024). Transport of CLCA2 to the nucleus by extracellular vesicles controls keratinocyte survival and migration. J. Extracell. Vesicles 13, e12430. 10.1002/jev2.12430 38602325 PMC11007793

[B21] Sharma M. Marin M. Wu H. Prikryl D. Melikyan G. B. (2023). Human immunodeficiency virus 1 preferentially fuses with pH-Neutral endocytic vesicles in cell lines and human primary CD4^+^ T-Cells. ACS Nano 17, 17436–17450. 10.1021/acsnano.3c05508 37589658 PMC10510587

[B22] Stein B. S. Gowda S. D. Lifson J. D. Penhallow R. C. Bensch K. G. Engleman E. G. (1987). pH-independent HIV entry into CD4-positive T cells *via* virus envelope fusion to the plasma membrane. Cell 49, 659–668. 10.1016/0092-8674(87)90542-3 3107838

[B23] van den Ende J. Defourny K. A. Y. Rabouw H. H. Tanenbaum M. E. Wubbolts R. W. Nolte-'t Hoen E. N. M. (2026). Development of a live-cell imaging assay to elucidate spatiotemporal dynamics of extracellular vesicle fusion with target cells. J. Extracell. Vesicles 15, e70228. 10.1002/jev2.70228 41764601 PMC12949999

[B24] van der Kant R. Fish A. Janssen L. Janssen H. Krom S. Ho N. (2013). Late endosomal transport and tethering are coupled processes controlled by RILP and the cholesterol sensor ORP1L. J. Cell Sci. 126, 3462–3474. 10.1242/jcs.129270 23729732

[B25] van Niel G. D'Angelo G. Raposo G. (2018). Shedding light on the cell biology of extracellular vesicles. Nat. Rev. Mol. Cell Biol. 19, 213–228. 10.1038/nrm.2017.125 29339798

[B26] Varkouhi A. K. Scholte M. Storm G. Haisma H. J. (2011). Endosomal escape pathways for delivery of biologicals. J. Control Release. 151, 220–228. 10.1016/j.jconrel.2010.11.004 21078351

[B27] Welsh J. A. Goberdhan D. C. I. O'Driscoll L. Buzas E. I. Blenkiron C. Bussolati B. (2024). Minimal information for studies of extracellular vesicles (MISEV2023): from basic to advanced approaches. J. Extracell. Vesicles 13, e12404. 10.1002/jev2.12404 38326288 PMC10850029

[B28] Zhuang Y. Guo X. Razorenova O. V. Miles C. E. Zhao W. Shi X. (2024). Coaching ribosome biogenesis from the nuclear periphery. bioRxiv. 06.21, 597078. 10.1101/2024.06.21.597078 38948754 PMC11212990

[B29] Zila V. Margiotta E. Turoňová B. Müller T. G. Zimmerli C. E. Mattei S. (2021). Cone-shaped HIV-1 capsids are transported through intact nuclear pores. Cell 184, 1032–1046 e1018. 10.1016/j.cell.2021.01.025 33571428 PMC7895898

